# TIMSS 2011 Student and Teacher Predictors for Mathematics Achievement Explored and Identified via Elastic Net

**DOI:** 10.3389/fpsyg.2018.00317

**Published:** 2018-03-15

**Authors:** Jin Eun Yoo

**Affiliations:** Department of Education, Korea National University of Education, Cheongju, South Korea

**Keywords:** machine learning, elastic net, regularization, penalized regression, TIMSS, mathematics achievement

## Abstract

A substantial body of research has been conducted on variables relating to students' mathematics achievement with TIMSS. However, most studies have employed conventional statistical methods, and have focused on selected few indicators instead of utilizing hundreds of variables TIMSS provides. This study aimed to find a prediction model for students' mathematics achievement using as many TIMSS student and teacher variables as possible. Elastic net, the selected machine learning technique in this study, takes advantage of both LASSO and ridge in terms of variable selection and multicollinearity, respectively. A logistic regression model was also employed to predict TIMSS 2011 Korean 4th graders' mathematics achievement. Ten-fold cross-validation with mean squared error was employed to determine the elastic net regularization parameter. Among 162 TIMSS variables explored, 12 student and 5 teacher variables were selected in the elastic net model, and the prediction accuracy, sensitivity, and specificity were 76.06, 70.23, and 80.34%, respectively. This study showed that the elastic net method can be successfully applied to educational large-scale data by selecting a subset of variables with reasonable prediction accuracy and finding new variables to predict students' mathematics achievement. Newly found variables via machine learning can shed light on the existing theories from a totally different perspective, which in turn propagates creation of a new theory or complement of existing ones. This study also examined the current scale development convention from a machine learning perspective.

## Introduction

Apparently, the Lee Sedol vs. AlphaGo match last year shocked the world; the 18-time world champion was defeated by AI (Artificial Intelligence). Although AI had beaten human champions in chess and Jeopardy (a game show), in 1997 and 2011, respectively, the game of “Go” had been considered formidable for AI to conquer, partly due to the game's close to infinite number of cases. Simply speaking, the recent triumphs of AI such as AlphaGo are made possible primarily by machine learning techniques, which is training machines (computers) to learn through algorithms as well as data, and therefore to search for optimum solutions. Specifically, AlphaGo used policy and value networks of neural networks as well as Monte Carlo tree search algorithms to find the next best move within given time frame and ultimately to win the game (Silver et al., 2016).

However, the machine learning method, neural networks, is infamous for its overfitting problems, as parameters can be exponentially increasing with multiple layers. Regularization techniques control the growth of coefficients and thus are used to solve overfitting problems. Regularization can be carried out with penalized regression in statistics. Penalized regression techniques such as Ridge, LASSO (Least Absolute Shrinkage and Selection Operator), and elastic net have been widely applied to various fields of study including computer science/engineering (Keerthi and Shevade, 2007; Sun et al., 2012; Wang et al., 2013; Youyou et al., 2015; Zhou et al., 2015; Shen et al., 2016), biology/medicine (He and Lin, 2010; Nie et al., 2010; Yang et al., 2010b; Li et al., 2011; Waldmann et al., 2013; Wang et al., 2017a), and finance (Kim and Swanson, 2014; Wu et al., 2014; Borke, 2017; Wang et al., 2017b).

Penalized regression has been popular with big data analyses, especially in situations where there are many variables and few observations, so-called “large p, small n” problems (Schäfer and Strimmer, 2005; Zou and Hastie, 2005; Zhao et al., 2009; He and Lin, 2010; Waldmann et al., 2013). However, large-scale educational data such as TIMSS (Trends in International Mathematics and Science Study) can also benefit from penalized regression with its hundreds of variables and thousands of participants. Previous TIMSS studies employed few indicators in their models selected based on theories and literature review, although TIMSS provides hundreds of student and teacher variables which have been collected after multiple experts' evaluations also along with theories and literature review. This may be partly due to the fact that conventional statistical methods have difficulty handling hundreds of variables in one model, resulting in convergence and/or overfitting problems. Moreover, many TIMSS research focused on some student variables only, and a handful of studies dealt with student and teacher variables in one statistical model, although it is a well-accepted fact that teachers play a crucial role in students' performance.

Therefore, throwing the hundreds of TIMSS student and teacher variables in one model and selecting variables with machine learning techniques can shed light on the existing theories and literature, especially if not yet investigated variables are found to be important via this approach. For instance, this study newly found students' internet connection at home, car possession at home, and teacher specialization in language/reading as predictors for mathematics achievement. Teacher variables such as collaboration in planning and preparing instructional materials and parental support perceived by teachers were not also frequently investigated in previous research.

According to a systematic review on TIMSS studies by Drent et al. (2013), there had been scant grade four studies, and most studies were on western or top-performing countries. Particularly, Korea has been one of the top-performing countries, but was not one of the well-studied countries (Drent et al., 2013). As an attempt to fill the gap in research, this study explored student and teacher variables relating to students' mathematics achievement, using Korean 4th graders and their teachers as the sample.

To reiterate, the main goal of this study was to find a prediction model for students' mathematics achievement out of hundreds of TIMSS student and teacher variables. Elastic net, the selected machine learning technique in this study, handled the inevitable non-convergence and overfitting problems resulting from considering hundreds of variables in one statistical model as well as multicollinearity problems commonly encountered in social science data. Relatedly, applying machine learning techniques such as elastic net to educational large-scale data also has implications to the current scale development convention, as items do not need to be parceled primarily for scale development and data reduction purposes.

In the next section, penalized regression methods including elastic net are explained in more detail along with bias-variance tradeoff, other variable selection methods, and cross-validation. Before directly moving to elastic net, ridge, and LASSO are introduced as its predecessors, and their limitations are discussed as well. In a nutshell, elastic net is preferable to ridge and LASSO for its variable selection feature and its strength in multicollinearity problems, respectively, and therefore was the selected machine learning technique of this study to analyze the TIMSS data.

## Regularization

### Bias-variance trade-off

In regression, the primary goal is to find the coefficient estimate close to the parameter. MSE (mean squared error) is used to evaluate this goal. Another goal which has not yet received deserved attention particularly in social sciences would be to find a model which fits future observations well. PE (Prediction Error) serves for evaluating this second goal, and is a well-documented topic in statistics (Hastie et al., 2009). PE comprises MSE and irreducible error. As the “irreducible” error is literally irreducible, attention is paid to variance and squared bias, the two components of MSE.

Notably, bias and variance trade-off each other. When a model becomes more complex, it picks up local structures of the data, and the bias of the coefficients gets lower, but the variance gets higher. As a result, overfitting may occur. In contrast, a simpler model increases the bias, but decreases the variance. Conventional ordinary least squares (OLS) or maximum likelihood (ML) methods have focused on obtaining unbiased estimates and lowering the variance among the unbiased estimates. On the other hand, regularization techniques focus on decreasing the overall MSE, by finding biased but lower-variance estimates.

### Variable selection

Variable selection is an important issue in data analysis, especially when there are many variables. Variable selection methods such as best-subset selection, forward selection, and backward elimination have been used for model construction, but they have weaknesses, compared to penalized regression methods (Hastie et al., 2009). To be more specific, best-subset can be applied to data with no more than 30–40 variables, and backward elimination has difficulty in dealing with the “large p, small n” problems. Both forward selection and backward elimination as discrete methods lack stability in model construction, as they either include or remove a variable one by one.

On the other hand, penalized regression methods, also called as shrinkage methods, continuously penalize coefficients with a regularization parameter. This continuous nature of penalized regression methods are known to produce more stable models than the aforementioned discrete methods. The most widely used penalized regression methods are ridge, LASSO, and elastic net.

### Ridge

Ridge was originally invented for multicollinearity problems (Hoerl and Kennard, 1970), but now is also well-known as an early penalized regression method. Suppose response variable y is estimated with *X* matrix of *N* observations and *P* predictors, and the X^T^X matrix is singular. Ridge puts an additional lambda value in the diagonals of the X^T^X matrix, and therefore the previously singular X^T^X matrix becomes invertible. Using the ridge regression, coefficients get shrunken due to the additional lambda.

(1) $$\hat{\beta }=argmin\left\{\frac{1}{2}\sum_{i=1}^{N}{\left(y_{i}\text{​}-\text{​}\beta_{0}\text{​}-\text{​}\sum_{j=1}^{P}x_{ij}\beta_{j}\right)}^{2}\text{​}+\text{​}\lambda \sum_{j=1}^{P}\beta_{j}^{2}\right\}$$

The penalty parameter, λ, determines the amount of regularization. It is easily shown that the λ value of 0 turns the equation into least squares estimation. In Equation (1), the first term in the right-hand side is ordinary least squares part, and the second term is the penalty function, which is called as L_2_ penalty. It is notable that ridge does not perform variable selection.

### LASSO

LASSO (Least Absolute Shrinkage and Selection Operator), invented by Tibshirani (1996), was designed to obtain a model of higher prediction accuracy and better interpretation than models from ordinary least squares methods. Compared to ridge, LASSO equation has a different penalty function, which is L_1_ penalty (Equation 2). While ridge sums up squared coefficients, LASSO uses the sum of absolute values. In a two-dimensional coefficients space, ridge's penalty constraint is shaped as a disk, and that of LASSO a diamond (Hastie et al., 2009). The error contours of L_1_ LASSO penalty has corners, and if the elliptical contours hit the corner, the corresponding coefficient becomes zero (Hastie et al., 2009). Therefore, let alone better interpretability, LASSO has the advantage of variable selection over ridge, which has huge practical implications especially in the “large p, small n” problems, frequently occurring in big data analyses.

(2) $$\hat{\beta }=argmin\left\{\frac{1}{2}\sum_{i=1}^{N}{\left(y_{i}\text{​}-\text{​}\beta_{0}\text{​}-\text{​}\sum_{j=1}^{P}x_{ij}\beta_{j}\right)}^{2}\text{​}+\text{​}\lambda \sum_{j=1}^{P}\left|\beta_{j}\right|\right\}$$

As was with ridge, the penalty parameter, λ, controls the amount of regularization. Larger values of λ shrink the coefficients more, and smaller values of λ makes the equation closer to least squares estimation. Unlike ridge, the estimation of LASSO does not provide closed forms, and therefore quadratic programming is required (Tibshirani, 1996).

### Elastic net

While LASSO is capable of variable selection, ridge is famous for its performance with multicollinearity (Zou and Hastie, 2005). Bridging between LASSO and ridge, elastic net exerts the advantages of the two, utilizing the L_1_ and L_2_ penalties of LASSO and ridge, respectively, in one equation. That is to say, elastic net not only selects variables, but also performs better than LASSO with collinear data (T. Hastie, personal communication, February, 9, 2017; R. Tibshirani, personal communication, February 1, 2017). As social science data such as TIMSS cannot be free from multicollinearity problems, particularly with its hundreds of variables, elastic net was the chosen method of this paper. The objective function of elastic net for Gaussian family is presented in Equation (3).

(3) $$\begin{array}{l} \hat{\beta }=argmin\{\frac{1}{2}\sum_{i=1}^{N}{\left(y_{i}-\beta_{0}-\sum_{j=1}^{P}x_{ij}\beta_{j}\right)}^{2}\\ \;+\lambda \sum_{j=1}^{P}(\alpha \left|\beta_{j}\right|+(1-\alpha )\beta_{j}^{2})\} \end{array}$$

More specifically, elastic net has two parameters, λ and α. The regularization (or penalty) parameter, λ, functions the same as with ridge and LASSO. The new tuning parameter, α, bridges between ridge and lasso. If α is 1, the equation equals to LASSO (equation 2), and α of 0 returns the ridge equation (Equation 1). Therefore, the value of α in-between 0 and 1 determines whether the model is closer to ridge or LASSO, taking advantage of both ridge and LASSO.

### Cross-validation (CV)

The penalty parameter, λ, determines the amount of regularization and thus model complexity. Choosing the right value of λ which minimizes prediction error is an essential part in penalized regression. K-fold cross-validation (CV) is a common approach to obtain the penalty parameter. K-fold CV partitions training data into K sets of equal size. K is typically chosen to be 5 or 10. For each fold (*k* = 1, 2, … *K*), the model is fitted with the training set excluding the k-th fold, and the fitted values are obtained for the k-th fold. This is repeated for every k-th fold, and each fold's CV error is calculated. The average of the K folds' CV errors serves as the overall CV error (Equation 4), and its standard error is also obtained.

(4) $$CV(\hat{f},\lambda )=\;\frac{1}{N}\sum_{i=1}^{N}L(y_{i,}\;{\hat{f}}^{-k\left(i\right)}(x_{i},\lambda ))$$

The best model which minimizes prediction error can be specified with CV, but the “one-standard-error rule” is typically employed to get the most parsimonious model (Hastie et al., 2009). This means that the least complex model is chosen among the models within one-standard error range of the best model. Aforementioned, a good model not only should fit the data well, but also fit new data well. In machine learning, a good model is obtained with “training” data, and evaluated with “test” (or an independent set of) data. Consequently, the corresponding λ is applied to the test data, and the model is evaluated with prediction accuracy, specificity, and sensitivity of the test data.

## Methods

### Data characteristics

In estimating students' academic achievement, TIMSS employs multiple imputation due to its matrix-sampling booklet design. Multiple imputation as a Bayesian approach draws multiple imputed (or plausible) values. TIMSS provides 5 PVs (plausible values) for mathematics and science as well as their subdomains, respectively. PVs are continuous, and TIMSS also provides categorical benchmarks for all respective PVs in 5 levels: 1 (Below Low; PVs < 400), 2 (Low; 400 < = PVs < 475), 3 (Intermediate; 475 < = PVs < 550), 4 (High; 550 < = PVs < 625), and 5 (Advanced; PVs > = 625).

Grade 4 student and teacher datasets of TIMSS 2011 Korea were merged, using the syntax codes of IEA's IDB Analyzer (Version 3.2.17). Although the original student dataset consisted of 4,334 students, the merged dataset had a total of 4,771 observations. The difference of 437 was due to the fact that two teachers of the 437 students responded to the teacher questionnaire. This study kept the first observation of the duplicates, which resulted in the original number of 4,334 observations with a total of 586 variables.

### Response variable

After merging, each student's mathematics class was created using all the five mathematics categorical benchmarks (ASMIBM01 to ASMIBM05) via majority vote, an ensemble method by Breiman (1996). For instance, if a student's benchmark variables were 1, 1, 2, 2, 1, then the student's class was coded as 1. Table 1 presents the majority vote results. There were 34 ties out of 4,334 Korean 4th graders. These ties were deleted from the subsequent analyses, and therefore the final sample size was 4,300.

**Table 1:** Majority vote result with 2011 TIMSS Korean 4th graders' math.

**Level**	**1**	**2**	**3**	**4**	**5**	**Ties**	**Total**
Observations	9	99	684	1,771	1,737	34	4,334

As about half of the students were in the “Advanced” level (5), the first four levels were collapsed, generating a new benchmark variable, ASMIBM. ASMIBM was coded with the criterion whether students reached “Advanced” (coded as “1”; 42%) or not (coded as “0”; 58%), and was used as the response variable of this study.

### Explanatory variables

Starting with the dataset of 586 variables on 4,300 4th graders and their teachers, irrelevant, or duplicate variables were removed and missing data was handled. Firstly, irrelevant variables relating to IDs (e.g., school ID, student ID, etc.), file maintenance (e.g., date of testing, file creation date, etc.), and weights (e.g., total student weight, etc.) were deleted from the explanatory variable pool. For duplicate variables such as students' gender (ITSEX, ASBG01) and birth information (e.g., ITBIRTHM, ASBG02A), variables of students' responses (e.g., ASBG01, ASBG02A) were removed.

Secondly, values such as omitted or invalid, logically not applicable, or not administered were marked as missing. The “omitted” responses came from the respondents' carelessness or unwillingness to answer the question. The “not applicable/administered” responses mainly resulted from the fact that TIMSS participating countries had great diversity in their educational systems. The missing rate of each variable was calculated, and 201 variables with their missing rates over 10% were removed from the dataset. This was a necessary step to maintain at least half of the original samples after listwise deletion.

Notably, except the newly created benchmark variable, ASMIBM, which served as the response variable of this study, all the other benchmark variables and PVs were excluded from the elastic net model. Inclusion of these academic performance variables would have dominated the model, which conveys little useful information to predict students' math achievement.

Among the explanatory variables, binary variables were dummy-coded so that girls and “Yes” responses (e.g., home possessions, etc.) were coded as 1; boys and “No” were coded as 0. Likert-like response variables such as number of books at home and computer use frequency were treated as continuous and coded as the original values.

### Cross-validation

After listwise deletion, this data cleaning process resulted in 2,353 4th graders (55% of the original data) with 163 student and teacher variables. For model validation, the observations were randomly split into training and test data sets with the conventional ratio of 7:3. The training data was used for model construction, and the test data was for model evaluation and generalization.

Particularly, the response variable, ASMIBM, was used as the stratifying variable to keep the rate of “Advanced” vs. “Others” in the training and test datasets. Table 2 presents numbers of students in each level for the training and test data sets.

**Table 2:** Training and test data.

	**1 (Advanced; 42%)**	**0 (Others; 58%)**
Data (*n* = 2,353)	995	1,358
Training data (*n* = 1,647)	696	951
Test data (*n* = 706)	299	407

Ten-fold CV (cross-validation) was used in this study. The training data were randomly split into 10-folds, and the model was fitted and evaluated using a range of λ values. The “one-standard-error rule” was employed for the most parsimonious model (Hastie et al., 2009), and the corresponding λ was identified and applied to the test data. Finally, the most parsimonious model was evaluated with prediction accuracy, specificity, and sensitivity of the test data.

**Table:** 

	Predicted as positive (Advanced)	Predicted as negative (Others)
Actual +	*TP* (True Positive)	*FN* (False Negative)
Actual –	*FP* (False Positive)	*TN* (True Negative)

The prediction accuracy was calculated as the sum of true positives and true negatives divided by the total. For instance, prediction accuracy of 70% indicates that the model correctly predicts 70 out of 100 new students' status (Advanced or Others). Sensitivity indicates the probability that a data point actually true is classified as true, and was calculated as true positives divided by the sum of true positives and false negatives $\left(=\frac{TP}{TP+FN}\right)$. Specificity indicates the probability that a data point actually false is classified as false, and was calculated as true negatives divided by the sum of false positives and true negatives$\left(=\frac{TN}{FP+TN}\right)$.

### Elastic net with logistic regression

As the response variable was dichotomous (G = 1; Advanced), a logistic regression model was utilized as the analysis model (Equation 5). As standardization of variables is necessary in penalized regression (Hastie et al., 2009), coefficients were estimated using Equation (6) after standardization.

(5) $$\log\frac{P(G=1\;|X=x)}{P(G=0|X=x)}=\beta_{0}+\beta^{T}x$$

(6) $$\begin{array}{l} \max\left\{\beta_{0}\epsilon R,\beta \epsilon R^{p}\right\}[\frac{1}{N}\sum_{i=1}^{N}logP\left(G_{i}|x_{i}\right)-\lambda \sum_{j=1}^{P}(\alpha |\beta_{j}|\\ \;+\left(1-\alpha \right)\beta_{j}^{2}/2)] \end{array}$$

All the programs were written in R 3.1.1. Specifically, the “glmnet” library was used (Friedman et al., 2017). The elastic net tuning parameter, α, was chosen as 0.5, as this value is known to perform well with correlated predictors (Hastie and Qian, 2016). To determine the penalty parameter, λ, a 10-fold CV was executed with cv.glmnet. The cv.glmnet package provides five types of measures for logistic regression models: misclassification error, AUC (Area Under the receiver operating characteristic Curve), binomial deviance, MSE (mean squared error), and MAE (mean absolute error).

Misclassification error is the proportion of misclassified cases among the total. AUC is the area under the ROC (Receiver Operating Characteristic) curve, and AUC of 1 indicates the perfect fit. Binomial deviance (or deviance) is a twice negative binomial log likelihood of the fitted model evaluated on the test data, and considered as an extension of the ordinary least squares' residual sum of squares in generalized linear models. MSE is the average of squared differences between actual and predicted values. MAE is the average of absolute differences between actual and predicted values. Compared to MAE, MSE penalizes large deviations more. AUC is compared to the baseline value of 0.5, and higher values of AUC indicate better model. All the other measures are interpreted the opposite; lower values of misclassification error, deviance, MSE, and MAE indicate better performance.

The steps for the coefficient estimation were as the following. First, this study used all the five measures. The λ value of each measure was determined using the “one-standard-error rule” (Hastie et al., 2009). Second, five models with the corresponding λ values from the first step were obtained. Their accuracy, sensitivity, and specificity results with the test data were compared, and the measure of the best prediction accuracy was selected. Lastly, elastic net coefficients were obtained, using the λ value in the previous step.

## Results

**Figure 1:**
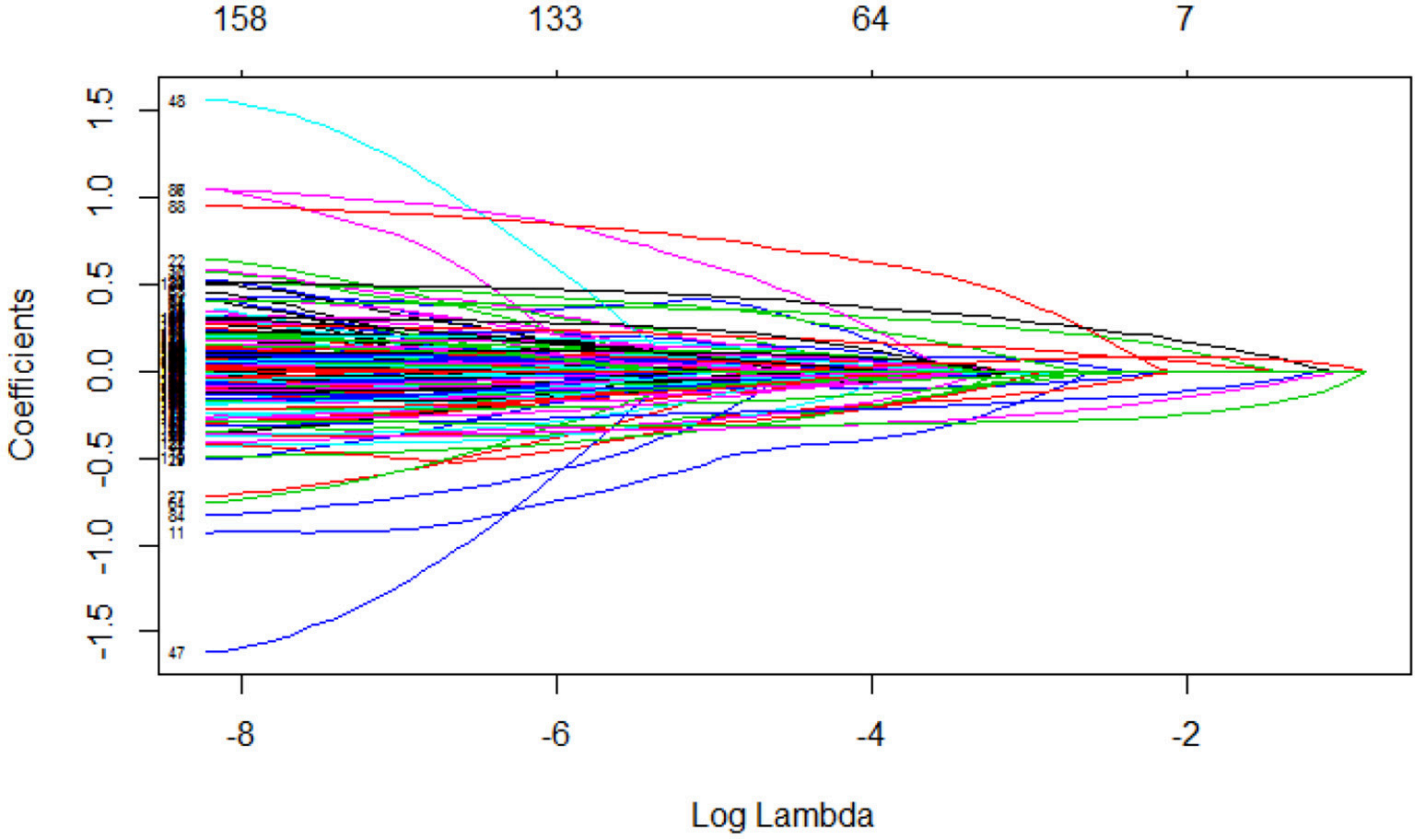
Regularization parameter (λ) and corresponding coefficients.

Figure 1 shows coefficients' paths with increasing values of λ, the regularization tuning parameter. Each curve corresponds to a predictor. The numbers above the box indicate the numbers of non-zero coefficients with the corresponding log(λ) values on the X-axis. The Y-axis indicates the coefficients of predictors. We can see that the coefficients get close to 0 with increasing λ.

**Figure 2:**
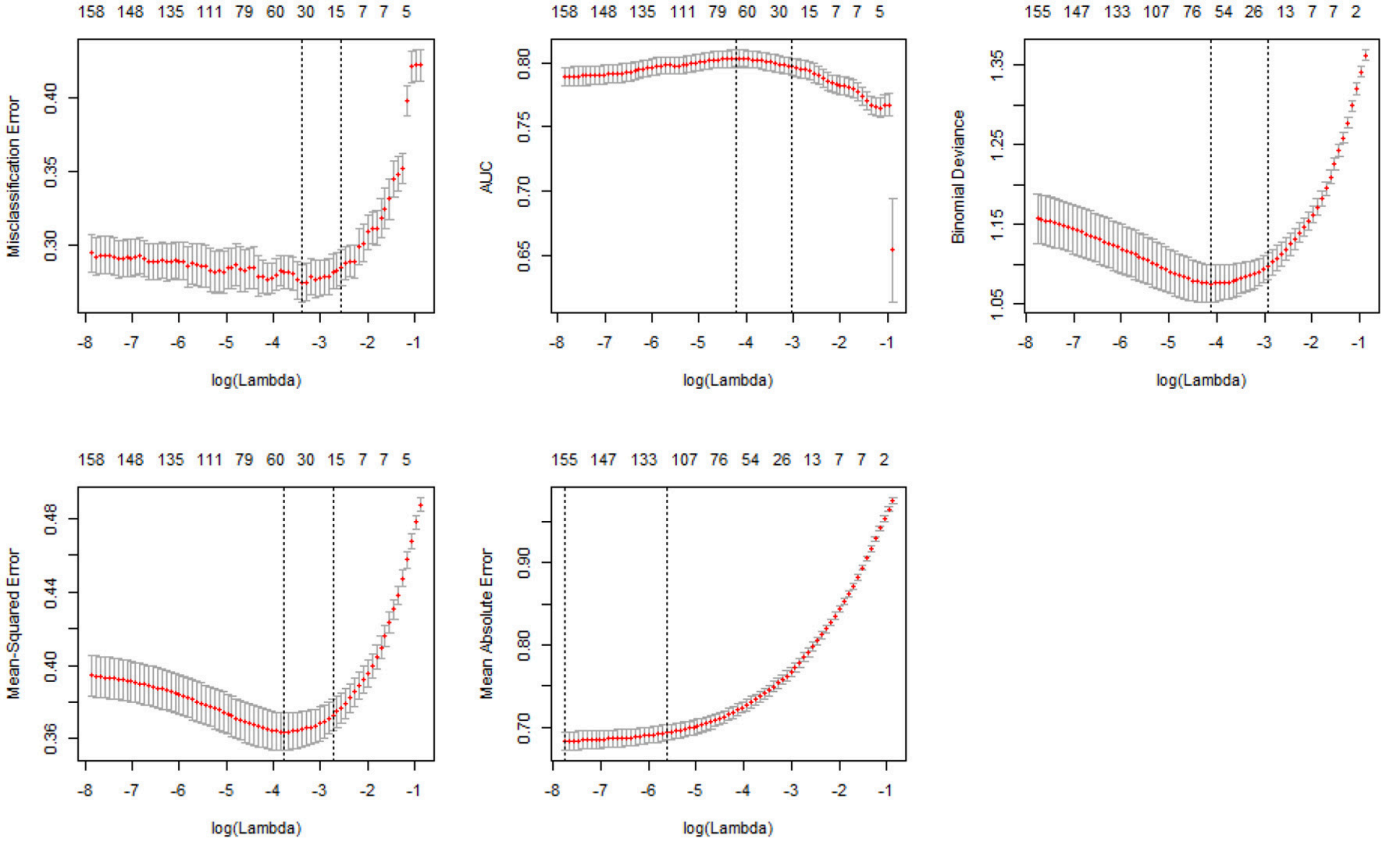
Ten-fold CV results of five measure types.

Figure 2 shows the 10-fold CV results of the five types of measures: misclassification error, AUC (Area Under Curve), deviance, MSE (Mean Squared Error), and MAE (Mean Absolute Error). As was with Figure 1, the numbers above the box indicate the numbers of nonzero coefficients with their corresponding log(λ) values on the X-axis. The Y-axis is in the unit of each measure. With all the measures except AUC, a lower value on the Y-axis indicates better performance. The vertical dotted lines in each plot are the upper and lower bounds of the one-standard-error rule. For instance, the fourth plot in Figure 2 shows the MSE result, and the number of nonzero coefficients with the upper bound [larger log(λ)] corresponds to 17. Therefore, a total of 17 variables were selected for the most parsimonious model with the one-standard-error rule using MSE. Among the five measures, MSE and misclassification error yielded the more parsimonious models, and MAE resulted in the least parsimonious model.

Table 3 presents prediction accuracy, sensitivity, and specificity results using the regularization parameter (λ) values of the 10-fold CV based on each measure. Each measure's number of variables and log(λ) were shown in Figure 2. Among the five measures, MSE showed the best performance, considering accuracy, sensitivity, and specificity of the test data as well as model parsimony. The regularization parameter, λ, was 0.0648 with the one-standard-error rule. This means that the MSE of the selected λ was within one standard error of the minimum value for the most regularized model. As results, 12 student and 5 teacher variables were identified out of the 162 predictors. The prediction accuracy, sensitivity, and specificity of the elastic net model with the test data were 76.06, 70.23, and 80.34%, respectively (Table 3).

**Table 3:** Prediction accuracy, sensitivity, and specificity of five measures.

	**Accuracy**	**Sensitivity**	**Specificity**
Misclassification	73.23	56.67	84.39
AUC	73.83	59.07	83.77
Deviance	73.75	60.00	83.02
MSE	76.06	70.23	80.34
MAE	73.75	63.15	80.90

Table 4 listed coefficients of selected variables from the elastic net model. Notably, penalized regression focuses on decreasing overall MSE by sacrificing the 'unbiasedness' of estimates. As the estimates of penalized regression are biased, the variance no longer equals to MSE. Although standard errors can be obtained via bootstrapping, standard errors only partially contribute to the MSE of penalized regression due to the biased estimates. As precise estimation of bias is nearly impossible with penalized regression (Goeman et al., 2016), precise estimation of MSE is also impossible. Therefore, it is typical that standard errors of coefficients are not provided with penalized regression.

**Table 4:** Selected variables, their labels, scales, and coefficients.

	**Variable**	**Label**	**Scale**	**Coefficient**
	Intercept			−1.262
1	ATBG05BC	Specialization/language-reading	1 (yes), 0 (no)	−0.060
2	ATBG06E	Parental support	1 (very high) to 5 (very low)	−0.060
3	ATBG06F	Parental involvement	1 (very high) to 5 (very low)	−0.005
4	ATBG07A	Sch/safe neighborhood	1 (agree a lot) to 4 (disagree a lot)	−0.098
5	ATBG10B	Interactions t/collaborate	1 (never or almost never) to 4 (daily or almost daily)	0.042
6	ASBG04	Amount of books in your home	1(0–10), 2(11–25), 3(26–100), 4(101–200), 5(200+)	0.214
7	ASBG05E	Internet connection	1 (yes), 0 (no)	0.287
8	ASBG05F	Car possession	1 (yes), 0 (no)	0.003
9	ASBG06A	How often use computer home	1 (everyday) to 4 (never)	−0.004
10	ASBM02C	Teacher is easy to understand	1 (agree a lot) to 4 (disagree a lot)	−0.030
11	ASBM03A	Usually do well in math	1 (agree a lot) to 4 (disagree a lot)	−0.230
12	ASBM03B	Harder for me than for others	1 (agree a lot) to 4 (disagree a lot)	0.255
13	ASBM03C	Just not good in math	1 (agree a lot) to 4 (disagree a lot)	0.103
14	ASBM03D	Learn quickly in mathematics	1 (agree a lot) to 4 (disagree a lot)	−0.024
15	ASBM03E	Good at working out problems	1 (agree a lot) to 4 (disagree a lot)	−0.163
16	ASBGSCM	Math self-confidence (score)		0.070
17	ASDGSCM	Math self-confidence (index)	1 (confident) to 3 (not confident)	−0.287

*Teacher variables were followed by student variables, and variables were presented in the order of TIMSS questionnaires*.

As expected, students' math self-confidence was a crucial factor to their math achievement (Table 4). Out of the 12 student variables, 7 variables related to students' math self-confidence. Specifically, the first five items measuring students' math self-confidence (ASBM03A to ASBM03E) were selected such as “I usually do well in mathematics,” “Mathematics is harder for me than for many of my classmates,” “I am just not good at mathematics,” “I learn things quickly in mathematics,” and “I am good at working out difficult mathematics problems.” The other two items in the same scale (ASBM03F and ASBM03G), “My teacher tells me that I am good at mathematics,” and “Mathematics is harder for me than any other subject” were not included in the model. The corresponding self-confidence composite score (ASBGSCM) and composite index (ASDGSCM) variables were also included in the elastic net model. One item on students' engagement in math lessons was selected: “My teacher is easy to understand” (ASBM02C). The more positively students responded to these items, the more likely they achieved the Advanced level.

The remaining four student variables were about home resources including book possession (ASBG04), internet connection (ASBG05E), car possession (ASBG05F), and computer usage (ASBG06A) at home. The more books the students had at home or the more often they used computer at home, the more likely they achieved the Advanced level. Interestingly, students who had a car at home or internet connection at home performed better than those who did not. Particularly, the internet connection and students' math self-confidence index variables had the highest coefficients among the 17 selected variables to predict students' math achievement, followed by individual self-confidence items, and amount of books at home.

Aforementioned, five teacher variables were selected. Specialization in language/reading (ATBG05BC) was the only variable in the teacher background section (“About You”). Students whose teacher specialized in language/reading had lower chance of achieving the Advanced level, but math or science specialization was not included in the model. Three variables were selected out of the school characteristics (“About Your School”). High levels of parental support for student achievement (ATBG06E) or parental involvement in school activities (ATBG06F) perceived by teachers positively related to students' achievement. The more the teachers agreed with the statement that their school was located in a safe neighborhood (ATBG07A), their students had a higher chance of achieving the Advanced level. Particularly, the safe neighborhood variable had the highest coefficient among teacher variables, followed by parental support. Lastly, one teacher variable on teacher interaction was selected. The more the teacher collaborated with other teachers in planning and preparing instructional materials (ATBG10B), the higher the students' performance was.

## Discussion

Conventional statistical techniques such as Hierarchical Linear Model (HLM) and Structural Equation Model (SEM) value theories. After theory-laden variables are identified, researchers design sampling strategies, collect data, run analyses, and interpret variables of statistical significance. This has been the typical process of quantitative research, which was valid and acceptable when study design and data collection cost considerable time and/or money. However, with the advent of so-called big data era, researchers have access to enormous amounts of data, without individually spending time and money for data collection. The primary questions left relate to how to analyze the data. Machine learning can be one of the solutions. Particularly, machine learning methodology has been gaining increasing interest in the eLearning community, as conventional statistical methodology carries clear limits to LMS (Learning Management Systems) types of data analysis (Guha, 2017; Pappas, 2017).

This foremost study showed the possibility of extending the new methodology to educational large-scale data, TIMSS 2011. Specifically, elastic net, a regularization method, was employed among the machine learning techniques. Social science research does not need to be confined to conventional methodology. Employing machine learning techniques provides methodological and practical advantages over conventional statistical methods including new variable exploration and identification without convergence problems. Implications of this study are discussed, followed by conclusion.

### Full use of data

First, this study made a full use of the wide range of TIMSS student and teacher data after data cleaning. This is virtually impossible with conventional methods such as HLM and SEM, as they fail to converge due to increasing number of parameters to be estimated. Partly related to this problem, previous research has been confined within a small set of variables or factors, depending on existing theories, statistical results, or a mix of the two. With machine learning and big (or large-scale) data, we may escape the researcher treadmill, and be able to find new important variables which have been ignored in the literature.

For instance, this study found internet connection and car possession at home were positively related to Korean 4th graders' math achievement. Particularly, car possession at home was a country-specific variable, and there was no TIMSS research using that variable as a predictor in math achievement. Internet connection, another home possession variable, was also rarely investigated as an individual predictor. Car possession and internet connection at home along with book possession and computer use at home are indicators to household income. This result corroborates the fact that students' household income or socio-economic status closely relate to their math achievement.

While frequently studied TIMSS student variables such as math self-confidence and amount of books at home were also identified in the elastic net model, often studied TIMSS teacher variables relating to differentiation/ adaptive instruction, curriculum quality, or class climate were not selected in the model. Likewise, all the five teacher variables of this study were not listed as significant factors for the 4th graders in the Drent et al. (2013)'s systematic review. The levels of safety at school and parental involvement perceived by teachers were included in the Drent et al.'s 8th grader results, though.

Among the five selected teacher variables, teacher collaboration in planning and preparing instructional materials appears to be relatively instantly implemented in practice, as this is something teachers can do on their end. In contrast, the other teacher variables such as parental support, parental involvement, and school neighborhood safety all perceived by teachers as well as specialization in major are not variables that can be readily changed. Therefore, studies on teacher collaboration in instructional materials should be furthered in order to improve students' math achievement.

Although this new approach can provide researchers with methodological breakthrough and novel insights, its inherently data-driven approach may seem inappropriate from the conventional view. There are ways to incorporate researchers' prior knowledge in regularization techniques such as elastic net. For instance, the R glmnet library has a penalty factor function (p.fac). One can easily set the penalty as “0” for variables of theoretic importance which should be included in the model.

### Scale development

Second and related to the first point, scale development may not be necessary with regularization methods such as elastic net. Aforementioned, increasing number of parameters to be estimated can result in convergence problems in conventional methods, and therefore data reduction has been a major issue in statistical research. Although item parceling has been popular for this matter in psychological studies, “to parcel or not to parcel” also has been under debate (Little et al., 2002, 2013; Yang et al., 2010a; Marsh et al., 2013).

In fact, scale development by item parceling can be troublesome. Item parceling prevents convergence problems by summing or averaging a set of items and making an index (or composite) variable. However, this practice of summing or averaging assumes that the set of items are from a unidimensional trait and are equally contributing to measurement of that trait. Thus, high reliability of the scale is a necessity, but it is not always the case in practice. For instance, TIMSS 2011 reported Cronbach's alphas of several scales such as “Students engaged in mathematics lessons” (Martin and Mullis, 2012). Unfortunately, around 30% of the participating countries had the Cronbach's alphas below 0.50, and the majority had the alphas around 0.60. The lowest alpha was that of Georgia, which was merely 0.21. This clearly violates the unidimensionality assumption required for scale development.

Another problem of item parceling relates to its differing item composition under the same label. That is to say, under the same labeling of a latent variable, different item parcels are often used depending on research. For instance, the latent variable, SES (Social Economic Status), is considered to be one of the most influential predictors to students' math achievement, and thus has been frequently studied in previous TIMSS research. However, different studies have used different combinations of items such as home resource items and parents' educational levels, although all the studies claimed that they indirectly measured “SES.” Therefore, we have to be cautious in interpretation when item parcels are used.

One easy answer to these problems of item parceling is not-to-parcel. The convergence problem resulting from not-to-parcel can be solved by regularization methods such as elastic net. To reiterate, elastic net requires neither item-parceling for data reduction purposes, nor assumptions such as unidimensionality for item-parceling, but selects important variables out of hundreds of predictor candidates without convergence problems. Moreover, individual variable's effect is also identifiable with the selected variables' coefficients. That is to say, if an item is selected in the model with the highest coefficient among a set of items in a scale, then we can say that that item exerts the highest influence on prediction accuracy in the scale. This is not easily done with item parceling, as item parceling lumps individual items together.

Lastly, TIMSS item parceling is vulnerable to issues of missing data. TIMSS estimates scale scores for composite variables or indices such as students' self-confidence in math (ASBGSCM or ASDGSCM), if students responded to only two or more items in a given scale (P. Foy, personal communication, July 17, 2015). If there are missing responses, summing only available responses can result in different subsets of items and thus biased estimates (Schafer and Graham, 2002). To reiterate, different sets of variables used for scale score estimation, depending on the missingness patterns, may result in biased estimates.

Under this circumstance, listwise deletion is considered the better method than the available-data method (Allison, 2001). However, large-scale datasets such as TIMSS severely suffers from listwise deletion. Particularly, non-administered or non-applicable (NA) responses plague a number of TIMSS variables, especially from the teacher questionnaire. With listwise deletion, handling these NA responses as missing results in dramatic reduction in sample size and thus reduction in efficiency. This study removed variables of more than 10% missing, which retained about 55% of the original samples. If the missing rates were increased to 20 or 30%, only 15 and 7% of the original samples remained, respectively. Removing variables of more than 10% missing partly alleviated the sample size issues, but potentially important variables, especially in the teacher questionnaire, might have been removed as a result. Research should be furthered on missingness patterns, scale score estimation, and regularization methods such as elastic net, particularly in the context of educational large-scale data.

## Conclusion

Research in the field of education has not yet paid enough, due attention to the recent big data/ machine learning techniques. Particularly, this study was one of the first studies to analyze educational large-scale data via elastic net. There can be disagreements on what constitutes educational big data, but educational large-scale data tentatively can serve the purposes of machine learning with its hundreds of variables and thousands of participants.

This study aimed to explore and identify possible sets of predictors using elastic net, currently one of the most popular machine learning techniques. A logistic regression model was employed to predict TIMSS 2011 Korean 4th graders' math achievement. Among 162 TIMSS variables, 12 student and 5 teacher variables were selected in the elastic net model, and its prediction accuracy was 76.06%. This means that the elastic net model of only 17 variables successfully predicted new students' mathematics class with 76.06% accuracy. Furthermore, this study was able to identify new predictors not yet investigated in previous research with conventional statistical methods. This study intentionally analyzed Korean 4th graders to fill the gap in the current TIMSS literature. Further machine learning studies with other TIMSS samples will help accumulate knowledge on students' math achievement, and consequently contribute to increasing students' math achievement.

It should be noted that penalized regression techniques focus on model prediction, not statistical significance. Therefore, predictors selected in penalized regression might not be statistically significant (T. Hastie, personal communication, January 28, 2017; R. Tibshirani, personal communication, January 28, 2017). Yoo and Rho (2017) employed group LASSO on social science large-scale data, and 15 out of 338 predictors were selected in the model. For comparison purposes, they constructed another model consisting of 83 predictors, based on literature review. Surprisingly, their model of only 15 predictors defeated the model of 83 predictors almost by 10%P in terms of prediction accuracy, specificity, and sensitivity. However, not all the 15 selected predictors in the group LASSO model were statistically significant.

In conclusion, it appears necessary to explore new variables to predict students' academic achievement and to examine the scale development convention via a machine learning technique. Newly found variables via machine learning can shed light on the existing theories from a totally different perspective, which in turn propagates creation of a new theory or complement of existing ones.

## Author contributions

JY designed the study, cleaned the data, performed data analyses, and wrote the manuscript.

### Conflict of interest statement

The author declares that the research was conducted in the absence of any commercial or financial relationships that could be construed as a potential conflict of interest.
